# Recognition of Tieguanyin Oolong Tea Fermentation Stages Based on RGB-HSI Bimodal Deep Fusion

**DOI:** 10.3390/foods15213949

**Published:** 2026-11-03

**Authors:** Yingqi Xue, Yuyan Huang, Guirong Xiao, Jingjing Zeng, Minjie Xuan, Yongkuai Chen, Zhijia Zheng

**Affiliations:** 1 Key Laboratory of Spatial Data Mining and Information Sharing of Ministry of Education, National & Local Joint Engineering Research Center of Satellite Geospatial Information Technology, Fuzhou University, Fuzhou 350108, China; 13950178807@163.com (Y.X.); xiaogr@fzu.edu.cn (G.X.); 19370451804@163.com (J.Z.); xmj1175542054@163.com (M.X.); 2 The Academy of Digital China, Fuzhou University, Fuzhou 350108, China; 3 Institute of Digital Agriculture, Fujian Academy of Agricultural Sciences, Fuzhou 350003, China; 18759871027@163.com

**Keywords:** Tieguanyin, fermentation stage recognition, RGB images, hyperspectral imaging, multimodal fusion, deep learning

## Abstract

Accurate recognition of successive Tieguanyin fermentation stages is essential for consistent processing control; however, adjacent stages often exhibit highly similar appearance characteristics, making fine-grained discrimination difficult. Although RGB images and hyperspectral imaging (HSI) data capture complementary appearance and spatial–spectral information, effectively integrating these heterogeneous representations remains challenging. To address this issue, an RGB–HSI bimodal dataset comprising 695 paired samples was constructed, and a bimodal deep fusion network, termed TeaMFNet, was developed for fine-grained fermentation stage recognition. For feature fusion, a Bidirectional Cross-modal Fusion (BCF) module enabled bidirectional information exchange between sample-level RGB appearance representations and HSI spatial–spectral representations. Building on this fusion, a Residual Feature Alignment (RFA) module refined the BCF-enhanced HSI features through lightweight residual correction, using the RGB representation as a discriminative reference to improve the alignment of heterogeneous features in a shared discriminative space. Furthermore, a Stage Prototype Module (SPM) introduced prototype-based constraints to enhance intra-stage compactness and inter-stage separability. During training, a Modality Confidence Modulation strategy was applied to balance the contributions of the two modalities. Experimental results showed that TeaMFNet achieved an accuracy of 93.28% and a Macro-F1 score of 93.38% on the random sample-level test split, which were numerically higher than those of DMD by 1.14 and 1.91 percentage points, respectively. However, leave-one-source-out evaluation yielded accuracies ranging from 74.10% to 91.45%, indicating reduced performance under source shifts and the need for broader cross-source validation. Ablation studies further confirmed the effectiveness of BCF, RFA, and SPM. Overall, these results demonstrate the potential of RGB–HSI bimodal fusion for distinguishing visually similar Tieguanyin fermentation stages, providing a basis for further investigation of objective fermentation stage assessment and digital monitoring of tea processing.

## 1. Introduction

Tea is an important specialty agricultural product. As one of the six major categories of tea in China, oolong tea combines the fresh aroma of green tea with the mellow taste of black tea, with Anxi Tieguanyin being a representative example [1,2]. The primary processing of Tieguanyin includes plucking, withering, fermentation, fixation, rolling, and drying, among which fermentation plays a key role in the development of its characteristic quality attributes. Fermentation generally consists of alternating tumbling and aeration, during which tea leaves undergo mechanical collisions, continuous moisture loss, and enzymatic oxidation, resulting in progressive changes in leaf color, morphology, aroma, and chemical composition [3]. The degree of fermentation directly affects the quality of finished tea. Insufficient fermentation may result in inadequate aroma and flavor development, whereas excessive fermentation can cause undesirable phenomena such as reddened stems and leaves [4]. In current production practice, determining the appropriate fermentation endpoint still relies largely on tea processors’ empirical judgments based on visual inspection, aroma assessment, and tactile evaluation. Such judgments are easily influenced by operator experience, variations among fresh leaf batches, and environmental conditions, making stable and objective evaluation difficult [4,5]. Moreover, because the Tieguanyin fermentation process evolves continuously, the differences in leaf color, red leaf margins, brown spots, and leaf posture between adjacent stages are often subtle. Therefore, developing an objective, rapid, and stable method for recognizing Tieguanyin fermentation stages is important for promoting standardized and intelligent oolong tea processing.

With the development of nondestructive sensing technologies and deep learning, machine vision and spectroscopic techniques have been increasingly applied to tea processing state recognition [6,7]. Machine vision, particularly RGB imaging, can capture external characteristics of tea leaves, including color, texture, morphology, and surface features. Accordingly, previous studies have employed RGB image-based color-space features, convolutional neural networks, and traditional machine-learning methods to recognize black tea fermentation states or Tieguanyin fermentation stages [4,5,8]. For example, Dong et al. [9] combined color features with a random forest model to predict black tea fermentation quality, whereas Chen et al. [5] and Huang et al. [8] developed the TGY-EOSR and T-GSR models, respectively, for Tieguanyin fermentation stage recognition. In contrast, spectroscopic techniques, particularly hyperspectral imaging (HSI), provide complementary information related to internal physicochemical changes, such as moisture variation, pigment transformation, and polyphenol oxidation [10]. Previous studies have applied HSI in combination with partial least squares regression, backpropagation neural networks, and deep-learning methods to tea component prediction, quality evaluation, and fermentation degree determination [11,12]. Overall, RGB images are effective at capturing external appearance changes, whereas HSI data provide spatial–spectral responses associated with internal physicochemical variations. However, neither modality alone is sufficient to comprehensively characterize the continuous Tieguanyin fermentation process [13].

To overcome the limitations of single-modality sensing, multimodal fusion has increasingly been introduced into tea processing monitoring and quality evaluation [14]. For example, Li et al. [15] integrated hyperspectral imaging with olfactory visualization for green tea quality evaluation. Jin et al. [16,17] explored the fusion of near-infrared spectroscopy and computer vision for evaluating black tea fermentation degree and fermentation quality. Zhang et al. [18] similarly investigated the mid-level fusion of near-infrared spectroscopy and machine vision for discriminating black tea fermentation degrees. Ren et al. [19] further introduced deep learning to integrate near-infrared spectroscopy, electronic eye, electronic tongue, and electronic nose signals for comprehensive tea quality evaluation. Ouyang et al. [20] developed an image–spectrum multimodal attention network incorporating contrastive learning for black tea withering quality assessment, reflecting a transition from conventional feature-level fusion toward deeper cross-modal representation learning. For Tieguanyin fermentation stage recognition, Huang et al. [21] fused RGB and hyperspectral features at the intermediate level and combined them with support vector machine, long short-term memory, and gated recurrent unit models, showing that multimodal fusion generally outperforms single-modality models. Collectively, these studies confirm the complementarity of appearance and spectral information and highlight the potential of multimodal fusion for tea processing state recognition.

However, existing multimodal methods still have limitations in fully exploiting cross-modal complementarity. Firstly, many previous approaches fuse multimodal features through direct feature combination or intermediate-level concatenation [14,21]. Although these methods can exploit RGB and HSI information simultaneously, the two modalities are generally encoded independently and combined only at the fusion stage, lacking explicit modeling of the deep cross-modal relationships between RGB appearance features and HSI spatial–spectral features, and thus failing to fully exploit the complementary potential of the two heterogeneous modalities. Secondly, the two modalities may contribute unequally during RGB–HSI joint modeling. RGB images and hyperspectral data differ markedly in imaging mechanism, feature dimensionality, data distribution, and discriminative capability [22,23,24,25,26], and evidence from Tieguanyin fermentation recognition confirms that they differ in single-modality recognition performance, while their fusion further improves classification accuracy [21]. This indicates that the two modalities are complementary but unequal in discriminative contribution; consequently, under simple concatenation or direct joint training, the model tends to rely more heavily on the more discriminative modality, preventing the useful information in the weaker modality from being fully converted into classification gains.

In addition to these cross-modal modeling challenges, the continuous nature of Tieguanyin fermentation further complicates stage discrimination. Adjacent fermentation stages often exhibit highly similar appearance and spectral characteristics [5,8]. Conventional classification supervision primarily optimizes decision boundaries without explicitly constraining the distribution structure of fused features. This may lead to insufficient intra-stage compactness and ambiguous inter-stage decision boundaries, thereby increasing misclassification between neighboring stages. These limitations motivate a fusion framework that can enhance cross-modal information exchange, align heterogeneous representations, balance the contributions of both modalities, and improve the discrimination of adjacent fermentation stages.

Accordingly, this study proposes an RGB–HSI bimodal fusion network, named TeaMFNet (Tea Multimodal Fusion Network), for Tieguanyin fermentation stage recognition. Taking RGB images and hyperspectral data as inputs, TeaMFNet integrates bidirectional cross-modal fusion, residual feature alignment, and stage prototype constraints to progressively enhance complementary information exchange, heterogeneous feature alignment, and stage-level discriminability. During training, a modality confidence modulation strategy is additionally employed to balance the contributions of both modalities. The main contributions of this study are summarized as follows:

(1) An RGB–HSI bimodal dataset for the Tieguanyin fermentation process was constructed, covering six processing batches and six representative fermentation stages. Each paired sample consists of three RGB images and one hyperspectral data cube.
(2) A Bidirectional Cross-modal Fusion (BCF) module was designed to perform bidirectional feature updating on sample-level global representations, enabling information exchange between RGB representations and HSI spatial–spectral representations before final fusion.
(3) A Residual Feature Alignment (RFA) module was proposed to refine the BCF-enhanced HSI features through lightweight residual correction using the RGB representation as a discriminative reference. This design improves the alignment of heterogeneous features while preserving HSI spatial–spectral information.
(4) A Stage Prototype Module (SPM) was introduced to construct a learnable prototype for each fermentation stage and impose prototype-based constraints that enhance intra-stage compactness and inter-stage separability.

These four contributions, encompassing dataset construction, cross-modal feature interaction, heterogeneous feature alignment, and stage-level discriminative learning, establish a complete framework for RGB–HSI bimodal fermentation stage recognition. The following section describes the dataset, preprocessing procedures, TeaMFNet architecture, experimental settings, and model evaluation.

## 2. Materials and Methods

### 2.1. Experimental Design

Fresh leaves of Anxi Tieguanyin were used as the experimental material in this study. Data were collected from six processing batches in Gande Town, Anxi County, Fujian Province, China, on 21 and 22 April 2026. The fresh leaves originated from three tea gardens, with each tea garden supplying two processing batches. The DEM-derived mean elevations of the three sampling sites were 859.88, 582.58, and 504.59 m. These sites were designated as relatively high-, medium-, and low-elevation sources, respectively.

Although Tieguanyin fermentation progresses continuously, identifying operationally meaningful processing states can support fermentation assessment. Accordingly, this study adopted a six-class classification framework based on representative stages in the actual production procedure. Fresh leaves underwent alternating tumbling and aeration operations, and six representative stages (S0–S5) were selected. The stages included after the first tumbling (S0), before the second tumbling (S1), before the third tumbling (S2), 3 h after the third tumbling (S3), appropriate fermentation (S4), and excessive fermentation (S5). Stage labels were assigned by experienced tea processors based on leaf color, reddening at the leaf margins, leaf posture, and aroma characteristics. To ensure temporal consistency between the two modalities, RGB images and hyperspectral data were acquired immediately after each stage was identified.

**Figure 1:**
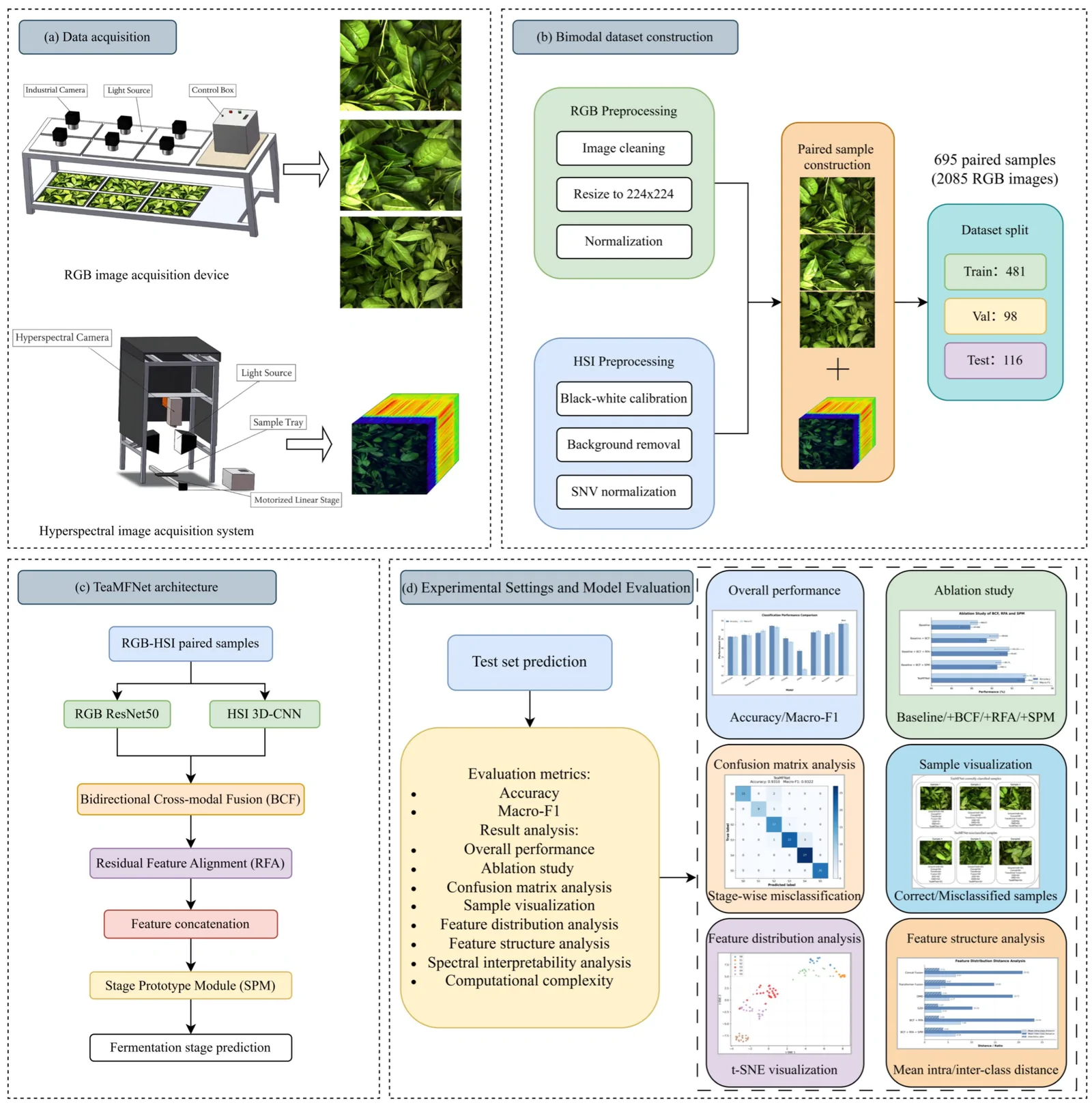
Experimental workflow of this study.

The experimental workflow comprises four components (Figure 1). RGB images and hyperspectral data were collected during the Tieguanyin fermentation process, and paired RGB–HSI samples were constructed through data preprocessing. On this basis, TeaMFNet was developed for fermentation stage recognition. Finally, TeaMFNet and the comparison models were trained under consistent settings and evaluated using common metrics.

### 2.2. Data Acquisition

A laboratory-built six-channel RGB image acquisition system (120 cm × 48 cm × 35 cm; Figure 1a) captured images during the Tieguanyin fermentation process. It consists of image acquisition, illumination, and sample transport modules. The image acquisition module is equipped with six industrial cameras (T-GE1000C-T-CL, Shenzhen Huateng Vision Technology Co., Ltd., Shenzhen, China), with a maximum image resolution of 3664 × 2748 pixels. The illumination module is equipped with six adjustable flat-panel light sources, each measuring 20 cm × 20 cm (JH-FLK200200, Dongguan Juhua Vision Technology Co., Ltd., Dongguan, China). During data acquisition, tea samples were placed on tea trays in the acquisition area for synchronous multi-angle imaging under stable illumination.

Hyperspectral data were acquired using a visible–near-infrared hyperspectral imaging system (Figure 1a). Its FX10 hyperspectral camera (SPECIM Spectral Imaging Ltd., Oulu, Finland) covers a spectral range of 400–1000 nm and contains 224 spectral bands. Other components include four 150 W halogen lamps (Wenzhou Aolijia Electric Appliance Co., Ltd., Wenzhou, China), a motorized translation stage, a lead-screw linear guide, a sample platform, and a computer. The system was warmed up before each acquisition to ensure data stability. During data acquisition, samples were evenly spread in a black container (35 cm × 24 cm × 3.5 cm), with the camera positioned 40 cm above the sample surface. The motorized stage performed line-scan imaging of the sample area, yielding one hyperspectral data cube per sample. Table 1 presents representative RGB images and hyperspectral data for fermentation stages S0–S5.

### 2.3. Bimodal Dataset Construction

RGB images and hyperspectral data were preprocessed separately. RGB images with blurring, abnormal exposure, severe occlusion, or background interference were removed, and three valid multi-view images were retained per sample for modeling. During training, random resized cropping was applied to generate 224 × 224 input images, followed by horizontal flipping, slight rotation, and color jittering for data augmentation. Validation and test images were resized to 224 × 224 pixels without random augmentation. All images were then normalized using the ImageNet mean and standard deviation.

For the hyperspectral data, white and dark reference calibration was applied to raw images to reduce camera dark-current noise and nonuniform illumination effects on spectral reflectance. The calibration formula is [27]:

(1) $$R=\frac{R_{0}-D}{W-D}$$

where R denotes the calibrated hyperspectral image, $R_{0}$ denotes the raw hyperspectral image, D denotes the dark reference image, and W denotes the white reference image.

After calibration, a region of interest containing tea leaves was cropped from each hyperspectral image, and surrounding backgrounds were removed. Cropped regions varied in size because leaf coverage differed among samples. They were resized to a uniform 96 × 96 pixels while retaining all 224 spectral bands, yielding 96 × 96 × 224 input cubes. Standard normal variate (SNV) transformation was applied to each spectrum by subtracting its mean and dividing by its standard deviation to reduce scattering effects and illumination-related variability.

The resulting dataset contained 695 valid RGB–HSI samples, each comprising three RGB images and one HSI cube, totaling 2085 RGB images and 695 HSI cubes. Table 2 summarizes sample counts for each fermentation stage.

Paired RGB–HSI samples were stratified by fermentation stage and split into training, validation, and test sets at a target ratio of approximately 70%:15%:15%. The final subsets contained 481, 98, and 116 paired samples, respectively, because stage-wise counts could not match the target ratio exactly. As this partition was performed at the sample level rather than by tea garden or processing batch, its results reflect recognition performance under the investigated sampling conditions rather than independent cross-source generalization.

### 2.4. RGB-HSI Bimodal Fusion Model

#### 2.4.1. Overall Framework

**Figure 2:**
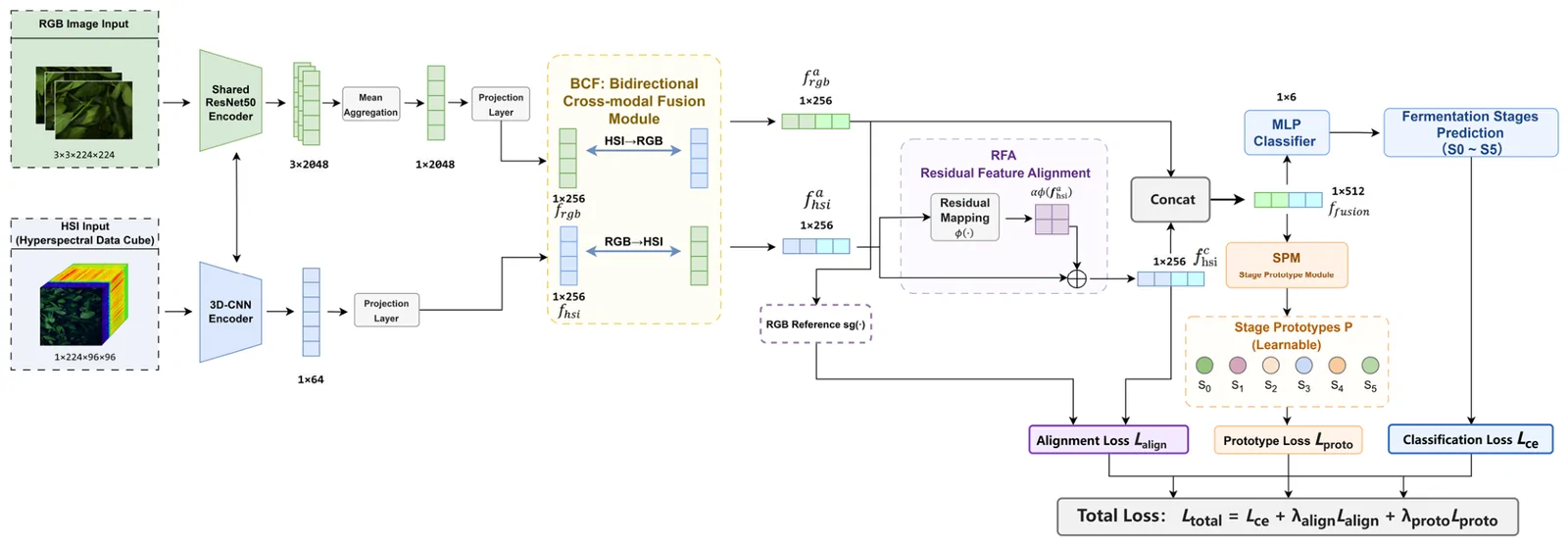
Overall architecture of TeaMFNet. Green and blue feature blocks represent RGB and HSI features, respectively.

The overall architecture of TeaMFNet is illustrated in Figure 2. The network mainly consists of an RGB image encoding branch, an HSI spatial–spectral encoding branch, a Bidirectional Cross-modal Fusion (BCF) module, a Residual Feature Alignment (RFA) module, a Stage Prototype Module (SPM), and a multilayer perceptron (MLP) classifier.

During forward propagation, the encoders extract modality-specific representations from their respective inputs. The BCF module performs bidirectional feature updating between the sample-level global RGB and HSI representations, facilitating the utilization of complementary information between the two modalities. The RFA module then applies residual cross-modal correction to the HSI features. The corrected HSI features are concatenated with RGB features to form the final fused features, which are passed through the MLP classifier to predict fermentation stages.

During model training, the fused features are also fed into the SPM to impose stage prototype constraints, thereby enhancing intra-stage compactness and inter-stage separability. TeaMFNet is jointly optimized using the feature alignment loss, stage prototype loss, and fused-feature classification loss. To mitigate potential learning imbalance between the two modalities, a Modality Confidence Modulation (MCM) strategy is further introduced to modulate gradient updates of both modality-specific encoders.

#### 2.4.2. Bimodal Feature Encoding Module

To improve the training stability of the bimodal model, the RGB and HSI unimodal models are first trained separately using only the training set, thereby obtaining unimodal pretrained weights for the two modality-specific encoders.

For each RGB–HSI paired sample, the RGB branch takes the three corresponding RGB images as inputs. Let $I_{i,j}$ denote the j-th RGB image of the i-th sample, and let K denote the number of RGB images associated with each sample, where K=3 in this study. A shared-parameter ResNet50 encoder $E_{rgb}\left(\cdot \right)$ [28] is used to extract deep visual features from each image, and the extracted features are averaged to obtain the sample-level RGB representation:

(2) $${\bar{g}}_{rgb,i}=\frac{1}{K}\sum_{j=1}^{K}E_{rgb}\left(I_{ij}\right)$$

Subsequently, the aggregated features are passed through a linear projection layer to obtain a fixed-dimensional RGB modality representation:

(3) $$f_{rgb,i}=P_{rgb}\left({\bar{g}}_{rgb,i}\right)$$

where $P_{rgb}\left(\cdot \right)$ denotes the RGB feature projection layer, and $f_{rgb,i}$ denotes the RGB feature used as input to the BCF module.

For the HSI encoding branch, the preprocessed hyperspectral data cube $X_{hsi,i}$ is fed into a 3D convolutional network-based HSI encoder $E_{hsi}\left(\cdot \right)$ to extract joint spatial–spectral features [29]. The extracted features are then projected by the HSI feature projection layer into a feature space with the same dimensionality as that of the RGB modality:

(4) $$f_{hsi,i}=P_{hsi}\left[E_{hsi}\left(X_{hsi,i}\right)\right]$$

where $P_{hsi}\left(\cdot \right)$ denotes the HSI feature projection layer, and $f_{hsi,i}$ denotes the HSI feature passed to the BCF module.

Through the encoding process described above, the RGB and HSI branches produce sample-level modality representations with the same feature dimensionality, providing the basic representations for the subsequent modules.

#### 2.4.3. Bidirectional Cross-Modal Fusion (BCF) Module

**Figure 3:**
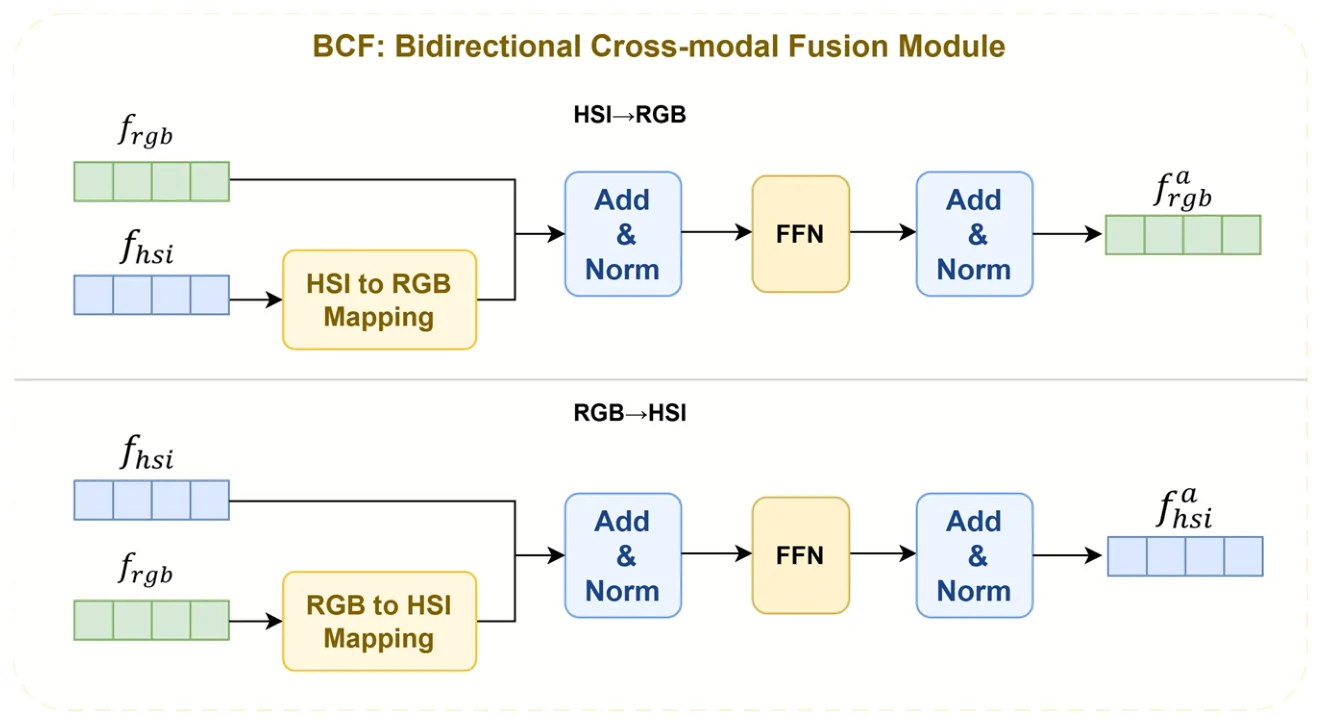
Architecture of the Bidirectional Cross-modal Fusion (BCF) module.

Previous studies on Tieguanyin fermentation degree recognition have shown that RGB image features and hyperspectral features exhibit different performance when modeled separately, whereas fusion models can further improve recognition accuracy [21]. This indicates that RGB appearance information and HSI spatial–spectral information are complementary for fermentation stage recognition. To exploit this complementarity, the BCF module takes the sample-level global representations generated by the two encoders as inputs and performs bidirectional cross-modal feature updating (Figure 3). Specifically, each modality representation is transformed and incorporated into the other modality through residual information injection, followed by nonlinear feature refinement. This process enables explicit information exchange between the two modalities before final fusion [30,31].

After bimodal feature encoding, the RGB and HSI representations of the i-th sample are denoted by $f_{rgb,i}$ and $f_{hsi,i}$, respectively. Both representations are projected into the same 256-dimensional feature space and expressed as single sample-level tokens. To enable information exchange before final fusion, each modality representation is transformed for updating the other modality and incorporated into the target representation through a residual connection, followed by layer normalization:

(5) $$z_{rgb,i}=\mathrm{LN}\left[f_{rgb,i}+\Phi_{hsi\to rgb}\left(f_{hsi,i}\right)\right]$$

(6) $$z_{hsi,i}=\mathrm{LN}\left[f_{hsi,i}+\Phi_{rgb\to hsi}\left(f_{rgb,i}\right)\right]$$

where $\Phi_{hsi\to rgb}\left(\cdot \right)$ and $\Phi_{rgb\to hsi}\left(\cdot \right)$ denote two independently parameterized cross-modal feature transformation functions for information transfer from HSI to RGB and from RGB to HSI, respectively. Each transformation is implemented using a multi-head attention layer operating on a single sample-level token. $LN\left(\cdot \right)$ denotes layer normalization, while $z_{rgb,i}$ and $z_{hsi,i}$ denote the intermediate representations after bidirectional cross-modal feature updating.

Subsequently, the intermediate representations are independently processed by modality-specific feed-forward networks, followed by residual connections and layer normalization to obtain the BCF-enhanced features [32]:

(7) $$f_{rgb,i}^{a}=\mathrm{LN}\left[z_{rgb,i}+{FFN}_{rgb}\left(z_{rgb,i}\right)\right]$$

(8) $$f_{hsi,i}^{a}=\mathrm{LN}\left[z_{hsi,i}+{FFN}_{hsi}\left(z_{hsi,i}\right)\right]$$

where $f_{rgb,i}^{a}$ and $f_{hsi,i}^{a}$ denote the BCF-enhanced RGB and HSI features, respectively.

#### 2.4.4. Residual Feature Alignment (RFA) Module

**Figure 4:**
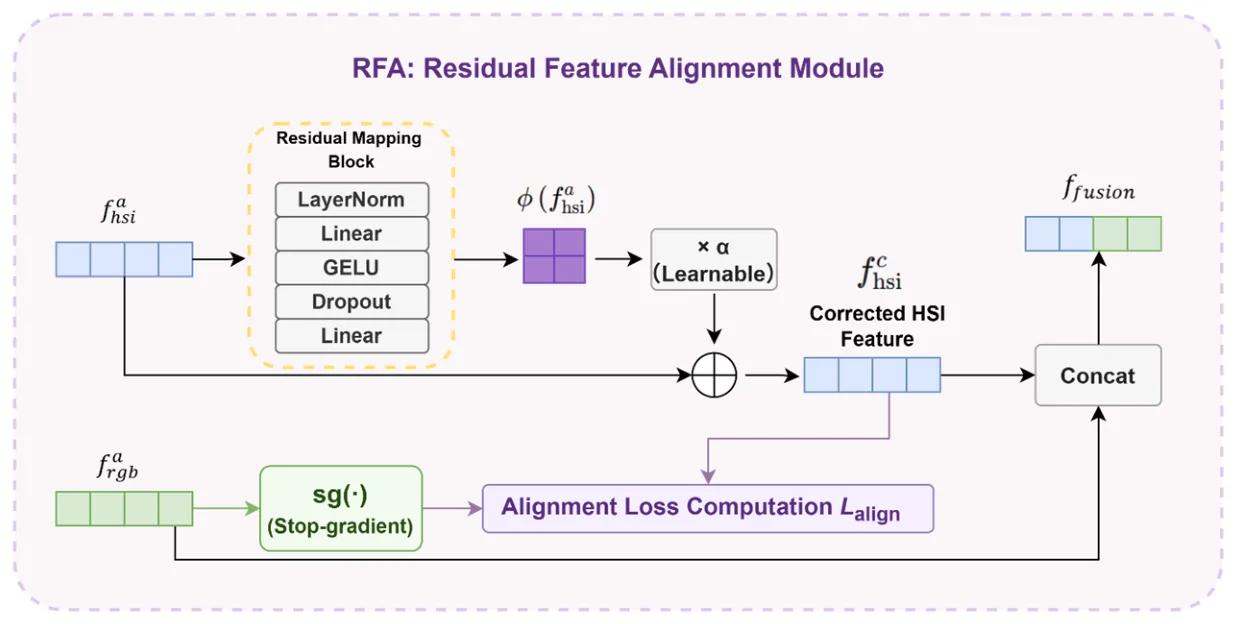
Architecture of the Residual Feature Alignment (RFA) module.

RGB and HSI features originate from different imaging mechanisms and therefore exhibit distinct feature distributions in the shared fusion space. Although the BCF module enables bidirectional information exchange between the RGB and HSI representations, it does not explicitly constrain the alignment of the two heterogeneous representations in the shared fusion space. Considering that RGB features directly capture stage-discriminative appearance cues such as leaf color, marginal reddening, brown spots, and leaf posture, the RFA module is designed to use the RGB representation as a discriminative reference and perform lightweight residual correction on the BCF-enhanced HSI representation. This design improves cross-modal feature alignment while preserving the spatial–spectral information of HSI, thereby enhancing the discriminability of the fused features [33,34]. The architecture of RFA is illustrated in Figure 4.

Let $f_{rgb,i}^{a}$ and $f_{hsi,i}^{a}$ denote the RGB and HSI features of the i-th sample after BCF interaction, respectively. The RFA module employs a residual mapping function $\phi \left(\cdot \right)$ to correct the HSI representation [28]:

(9) $$f_{hsi,i}^{c}=f_{hsi,i}^{a}+\alpha \;\phi \left(f_{hsi,i}^{a}\right)$$

where $\phi \left(\cdot \right)$ consists of LayerNorm, a linear layer, a GELU activation function, Dropout, and another linear layer, and is used to learn a residual correction for the HSI representation in the shared feature space. α is a learnable residual scaling coefficient. In this study, α is initialized to zero, such that the BCF-enhanced HSI feature remains unchanged at initialization. During training, α is adaptively learned to control the scale of the residual correction. This design avoids excessive transformation of the HSI spatial–spectral representation, thereby improving cross-modal alignment while preserving its existing discriminative information.

To constrain the direction of residual correction, a feature alignment loss is introduced:

(10) $$L_{align}=\frac{1}{B}\sum_{i=1}^{B}{∥f_{hsi,i}^{c}-sg(f_{rgb,i}^{a})∥}_{2}^{2}$$

where B denotes the batch size, and $sg\left(\cdot \right)$ denotes the stop-gradient operation. The alignment loss treats the stop-gradient RGB representation as a fixed reference and minimizes the discrepancy between it and the corrected HSI representation in the shared feature space. Consequently, gradients from this loss update only the HSI-side correction pathway without affecting the RGB branch.

Finally, the BCF-enhanced RGB feature $f_{rgb,i}^{a}$ and the RFA-corrected HSI feature $f_{hsi,i}^{c}$ are concatenated to obtain the final fused feature $f_{fusion,i}$ (Equation (11)), which is subsequently fed into the classifier and the SPM for fermentation stage recognition.

(11) $$f_{fusion,i}=\mathrm{Concat}\left(f_{rgb,i}^{a},f_{hsi,i}^{c}\right)$$

#### 2.4.5. Stage Prototype Module (SPM)

**Figure 5:**
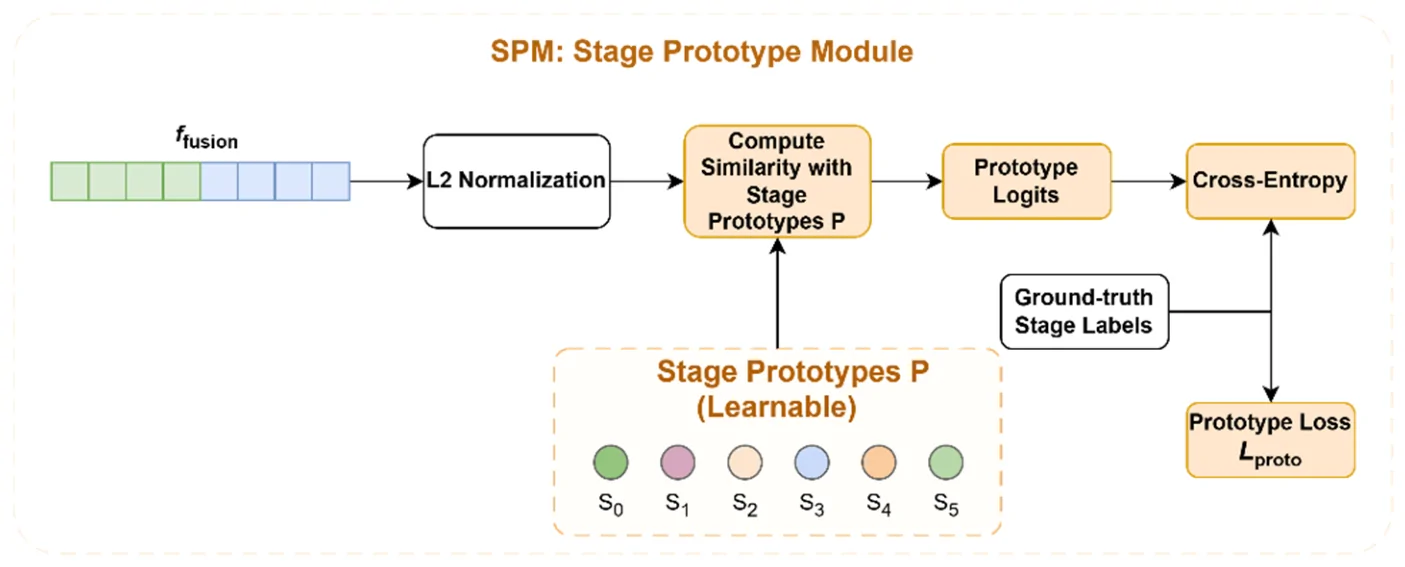
Architecture of the Stage Prototype Module (SPM). The green and blue segments of the fused feature vector represent RGB and HSI features, respectively.

The Tieguanyin fermentation process evolves continuously, and the appearance and spectral differences between adjacent stages are subtle. When supervised solely by cross-entropy loss, the model can learn class decision boundaries but does not explicitly constrain the feature distributions within the fused feature space. This may result in insufficient intra-stage compactness and ambiguous boundaries between adjacent stages. To enhance the stage discriminability of the fused features, we propose SPM, which explicitly introduces a learnable prototype for each fermentation stage as its class center in the fused feature space [35,36]. Unlike conventional classification that relies solely on classifier weights, these prototypes encourage the model not only to classify samples correctly but also to regularize the feature distribution itself, thereby enhancing intra-stage compactness and inter-stage separability [37]. The architecture of SPM is shown in Figure 5.

Let C denote the number of fermentation stages, where C=6 in this study, and let d denote the dimensionality of the fused features. The SPM defines a learnable prototype vector $p_{c}\in R^{d}$ for the c-th fermentation stage, which serves as a representative center for the corresponding stage in the fused feature space. These prototypes are jointly optimized alongside other network parameters through backpropagation. For the i-th sample, the scaled cosine similarities between its fused feature $f_{fusion,i}$ and all stage prototypes are calculated to obtain the prototype-based classification logits:

(12) $$z_{i,c}^{proto}=s\;\frac{f_{fusion,i}^{T}p_{c}}{{∥f_{fusion,i}∥}_{2}{∥p_{c}∥}_{2}},c=1,2,\ldots ,C$$

where s is a scaling coefficient used to scale the cosine-similarity logits before the Softmax operation. The prototype discrimination loss corresponding to SPM is defined as:

(13) $$L_{proto}=-\frac{1}{B}\sum_{i=1}^{B}\log\frac{\exp\left(z_{i,y_{i}}^{proto}\right)}{\sum_{c=1}^{C}\exp\left(z_{i,c}^{proto}\right)}$$

where B denotes the batch size, and $y_{i}$ denotes the true fermentation stage label of the i-th sample. This loss encourages each fused feature to exhibit a higher similarity to the prototype of its corresponding stage than to the prototypes of other stages.

#### 2.4.6. Modality Confidence Modulation (MCM) Strategy and Loss Functions

RGB images and HSI data differ in imaging mechanisms and discriminative capabilities, which may lead to inconsistent learning progress between the two modalities during joint bimodal training. To balance the contributions of both modalities, the MCM strategy is employed [38]. The strategy uses pretrained unimodal reference models to evaluate the classification confidence of the RGB and HSI modalities and preferentially updates the encoder of the modality with lower confidence during the early stage of training.

For modality $m\in \left\{rgb,hsi\right\}$, its classification confidence is defined as the average probability assigned to the true classes of the training samples by the corresponding unimodal reference model:

(14) $$\rho^{m}=\frac{1}{N}\sum_{i=1}^{N}p_{i}^{m}\left(y_{i}\right),m\in \left\{rgb,hsi\right\}$$

where N denotes the number of training samples used to estimate modality confidence, $y_{i}$ denotes the true fermentation stage label of the i-th sample, and $p_{i}^{m}\left(y_{i}\right)$ denotes the probability assigned to the true class $y_{i}$ by the unimodal reference model of modality m. Based on the values of $\rho^{rgb}$ and $\rho^{hsi}$, the modality with lower confidence is identified as the relatively weaker modality $m_{weak}$, while the other is identified as the stronger modality $m_{strong}$.

During the early stage of training, MCM preferentially updates the weaker-modality encoder while temporarily suppressing the gradient updates of the stronger-modality encoder. Both modality encoders are then jointly updated. For modality m, the gradient modulation coefficient at the e-th training epoch is defined as:

(15) $$\kappa_{m}\left(e\right)=\left\{\begin{array}{cc} 1, & m=m_{weak},e\leq \tau_{g},\\ 0, & m=m_{strong},e\leq \tau_{g},\\ 1, & e>\tau_{g}, \end{array}\right.$$

where $\tau_{g}$ denotes the number of training epochs in the weak-modality priority learning stage. Before the optimizer updates the network parameters, the gradient of each modality encoder is multiplied by its corresponding modulation coefficient $\kappa_{m}\left(e\right)$, and the encoder parameters are then updated using the modulated gradients. The modulation is applied only to the RGB and HSI encoders, while the BCF, RFA, SPM, and classifier are optimized normally according to the total loss. Through this strategy, the model preferentially strengthens the weaker modality during the early training stage and subsequently enables joint updating of both modality encoders.

TeaMFNet is jointly trained using the classification loss, feature alignment loss, and stage prototype loss. The total loss is defined as:

(16) $$L_{total}=L_{ce}+\lambda_{align}L_{align}+\lambda_{proto}L_{proto}$$

where $L_{ce}$ denotes the cross-entropy loss, $L_{align}$ denotes the feature alignment loss of the RFA module, and $L_{proto}$ denotes the stage prototype loss of the SPM. $\lambda_{align}$ and $\lambda_{proto}$ are the weighting coefficients for $L_{align}$ and $L_{proto}$, respectively.

### 2.5. Experimental Settings and Model Evaluation

This section presents experimental settings and model evaluation methods. All comparative experiments used identical dataset splits and evaluation metrics, with training configurations kept as consistent as possible for fair comparison.

The experiments were implemented in Python 3.10 using PyTorch 2.6.0 with CUDA 12.4. Training and testing were performed on an NVIDIA GeForce RTX 4090 GPU (24 GB memory). Table 3 summarizes the training configurations. All models used AdamW with an initial learning rate of $2\times 10^{-5}$, a weight decay coefficient of $1\times 10^{-4}$, a batch size of 4, and 20 training epochs. For MCM, the weak-modality priority learning stage was set to 10 epochs ${(\tau }_{g}=10$). The alignment and prototype loss weights were $\lambda_{align}=0.01$ and $\lambda_{proto}=0.1$, respectively, and the scaling factor s in the prototype-based classification head was set to 16. The loss weights and scaling factor were selected by evaluating multiple candidate values based on performance on the validation set. For the overall performance comparison, each model was independently trained with three random seeds (1, 2, and 42), and the mean and standard deviation were reported. Confusion matrices and t-SNE visualizations were generated from the seed-42 model. During training, the checkpoint with the highest validation Macro-F1 was selected for final test evaluation.

Accuracy and Macro-F1 were adopted as the evaluation metrics. Accuracy measures the overall classification performance, whereas Macro-F1 evaluates the average recognition performance across all classes. Accuracy is calculated as:

(17) $$Accuracy=\frac{N_{correct}}{N}$$

where $N_{correct}$ denotes the number of correctly predicted samples, and N denotes the total number of test samples. For class c, its F1-score is defined as:

(18) $$F1_{c}=\frac{2\times {Precision}_{c}\times {Recall}_{c}}{{Precision}_{c}+{Recall}_{c}}$$

(19) $${Precision}_{c}=\frac{TP_{c}}{TP_{c}+FP_{c}}$$

(20) $${Recall}_{c}=\frac{TP_{c}}{TP_{c}+FN_{c}}$$

where ${TP}_{c}$, ${FP}_{c}$, and ${FN}_{c}$ denote true positives, false positives, and false negatives for class c, respectively. Macro-F1 is then calculated as:

(21) $$Macro\text{-}F1=\frac{1}{C}\sum_{c=1}^{C}F1_{c}$$

Computational complexity and inference efficiency were assessed using parameter count, floating-point operations (FLOPs), GPU inference latency, and peak allocated GPU memory.

To examine whether the classification outputs retained information about the ordering of fermentation stages, a post-hoc analysis of the six-class Softmax probabilities (S0–S5) was conducted. For each test sample, a probability-weighted stage score is calculated as follows:

(22) $${\hat{s}}_{i}=\sum_{k=0}^{5}k\;p_{ik}$$

where $p_{ik}$ denotes the Softmax probability of stage $S_{k}$ for the i-th sample, and ${\hat{s}}_{i}$ represents the corresponding probability-weighted stage score. The predicted probability distributions were also analyzed to examine the probability mass assigned to the ground-truth stage, adjacent stages, and non-adjacent stages. The score served as an auxiliary indicator of stage ordering rather than a direct measure of continuous fermentation degree.

GradientSHAP was employed to quantify individual hyperspectral band contributions to TeaMFNet’s fermentation stage predictions [39,40]. The analysis was conducted on 116 paired test samples, using 24 training samples (four per stage) as the reference background. GradientSHAP was implemented using Captum 0.9.0 with 16 attribution samples and a noise standard deviation of zero. Attributions were calculated with respect to the ground-truth class fusion logits while keeping the RGB inputs unchanged. Wavelength importance was calculated by averaging absolute attribution values across spatial dimensions and then across samples.

## 3. Results

### 3.1. Comparison of Recognition Performance

#### 3.1.1. Comparison of Overall Recognition Performance

TeaMFNet was evaluated against eight representative fusion models for RGB–HSI bimodal Tieguanyin fermentation stage recognition, including Concat Fusion, LMF, Transformer Fusion, DMD, TCPFMC, EMOE, MultiEMO, and G2D. Concat Fusion directly concatenates RGB and HSI features into a joint representation and serves as the baseline for examining the fundamental complementarity between the two modalities. LMF captures high-order cross-modal interactions through low-rank tensor decomposition [41]. Transformer Fusion employs attention to model cross-modal relationships and adaptively emphasize informative modality features [30]. DMD separates modality-shared and modality-specific information, reducing inter-modal redundancy and improving the discriminability of fused features [42]. TCPFMC estimates modality or prediction reliability through credibility modeling to limit the influence of uncertain information [43]. EMOE fuses feature subspaces through expert selection or weighting mechanisms, enhancing the representation of heterogeneous multisource information [44]. MultiEMO employs bidirectional multi-head cross-modal attention to model inter-modal correlations [31]. G2D alleviates modality imbalance through unimodal teacher distillation and sequential modality prioritization, thereby reducing the suppression of the weaker modality [38]. Collectively, these models represent diverse fusion strategies and provide a representative comparison set.

Table 4 shows clear performance differences among the models. Concat Fusion, which relies solely on feature concatenation, achieved an accuracy of 86.22% and a Macro-F1 of 86.13%. These results indicate that although RGB images and HSI data provide complementary information, simple concatenation enables no explicit information exchange between the global RGB and HSI features. LMF and Transformer Fusion improved accuracy to 87.22% and 88.31% and Macro-F1 to 87.01% and 89.47%, respectively, suggesting that stronger feature interactions enhance bimodal representations. DMD performed strongly among the comparison models, achieving 92.14% accuracy and 91.47% Macro-F1, supporting the benefit of modality feature disentanglement for RGB–HSI bimodal recognition. G2D and MultiEMO also outperformed the basic Concat Fusion model, achieving accuracies of 88.58% and 87.56% and Macro-F1 scores of 89.38% and 88.44%, respectively. These results indicate that modality balancing and cross-modal attention modeling can enhance bimodal feature representation to some extent. TCPFMC and EMOE performed relatively poorly on this dataset, indicating that different fusion models vary in their adaptability to Tieguanyin fermentation stage recognition under conditions of limited sample size and subtle differences between adjacent stages.

TeaMFNet achieved an accuracy of 93.28% and a Macro-F1 score of 93.38%, which were numerically higher than those of DMD by 1.14 and 1.91 percentage points, respectively. Given the limited test-set size of 116 samples, these modest differences should be interpreted cautiously. Compared with Concat Fusion, the corresponding improvements were 7.06 percentage points and 7.25 percentage points. These results demonstrate that combining bidirectional cross-modal fusion, residual feature alignment, and stage prototype constraints enhances RGB–HSI feature representation and improves the discrimination of adjacent fermentation stages.

#### 3.1.2. Comparison of Recognition Performance Across Fermentation Stages

To further compare stage-wise recognition performance, five models were selected for analysis. Concat Fusion was chosen as the representative of basic concatenation-based fusion. Transformer Fusion, which models cross-modal interactions through attention, was included to examine the effect of explicit feature interaction on stage recognition. DMD and G2D were included as the strongest baselines from the comparative experiments, representing feature disentanglement and modality balancing, respectively. Together with TeaMFNet, these models cover the major fusion paradigms, enabling a clear stage-wise comparison.

**Figure 6:**
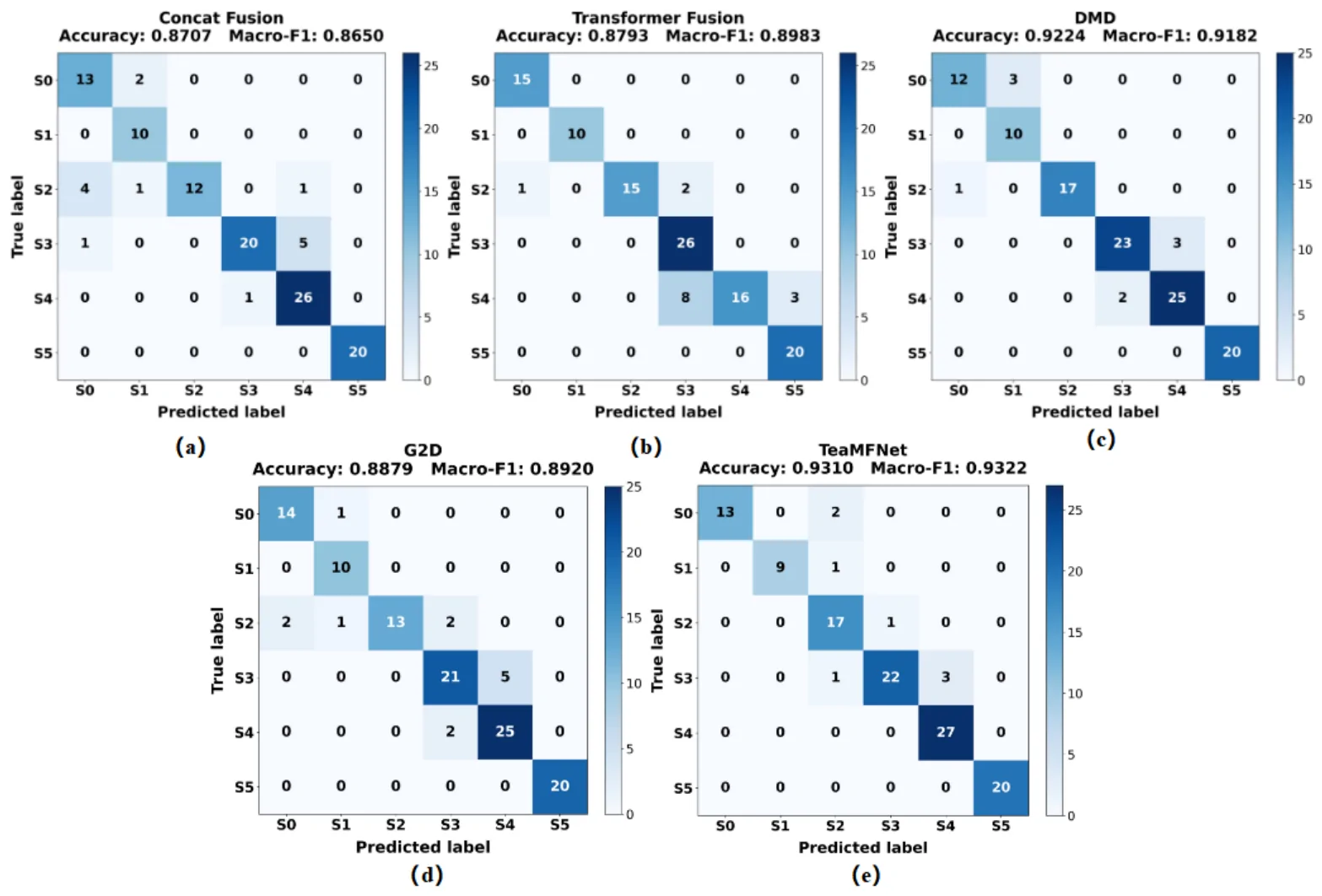
Comparison of confusion matrices of different fusion models: (**a**) Concat Fusion; (**b**) Transformer Fusion; (**c**) DMD; (**d**) G2D; and (**e**) TeaMFNet. All results were obtained from models trained with random seed 42.

The stage-wise recognition accuracy of each model is presented in Table 5. All models achieved an accuracy of 100.00% at S5, indicating that the excessive fermentation stage exhibits relatively distinctive characteristics and is easy to distinguish. Most models also performed well at S1, with Concat Fusion, Transformer Fusion, DMD, and G2D all reaching 100.00%. However, TeaMFNet misclassified one S1 sample as S2, suggesting similarity between adjacent early stages. Accuracy at the intermediate stages (S2, S3, and S4) varied markedly across models. Concat Fusion and G2D achieved accuracies of 66.67% and 72.22% at S2, respectively, whereas Transformer Fusion achieved only 59.26% at S4. These results indicate that the feature differences between adjacent stages are relatively subtle, making them more prone to misclassification. As further shown in Figure 6, several comparison models exhibited notably low accuracy at certain stages, whereas TeaMFNet maintained balanced performance across stages S0–S5. TeaMFNet achieved accuracies of 94.44%, 100.00%, and 100.00% at S2, S4, and S5, respectively, demonstrating more effective use of complementary RGB–HSI information for recognition across fermentation stages.

Figure 6a shows that Concat Fusion exhibited multiple misclassifications, particularly between adjacent stages such as S0–S1, S2–S3, and S3–S4. This indicates that although simple feature concatenation can utilize bimodal information, it does not enable explicit information exchange between the sample-level global RGB and HSI representations, and its ability to distinguish subtle differences between adjacent stages remains limited. Transformer Fusion produced predictions more concentrated along the diagonal than Concat Fusion (Figure 6b), indicating improved cross-modal interaction through attention, although confusion persisted at transition stages. DMD achieved relatively strong overall performance, suggesting that feature disentanglement improves fused-feature discriminability, but misclassifications remained at S0–S1 and S3–S4 (Figure 6c). G2D used modality balancing to alleviate strong-modality dominance and yielded diagonal-concentrated predictions, yet failed to eliminate adjacent-stage confusion (Figure 6d). Thus, despite improving overall performance, these models remained limited in resolving continuously evolving fermentation states with indistinct stage boundaries.

The confusion matrix of TeaMFNet shows predictions more concentrated along the diagonal, with few off-diagonal misclassifications (Figure 6e), indicating more stable recognition across fermentation stages. Its strong performance at the mid-to-late stages (S3, S4, and S5) suggests that it effectively captured stage-related discriminative cues such as leaf color, red leaf margins, brown spots, and leaf posture changes. The remaining misclassifications mainly occurred between adjacent stages, consistent with continuous fermentation and non-discrete stage boundaries.

**Figure 7:**
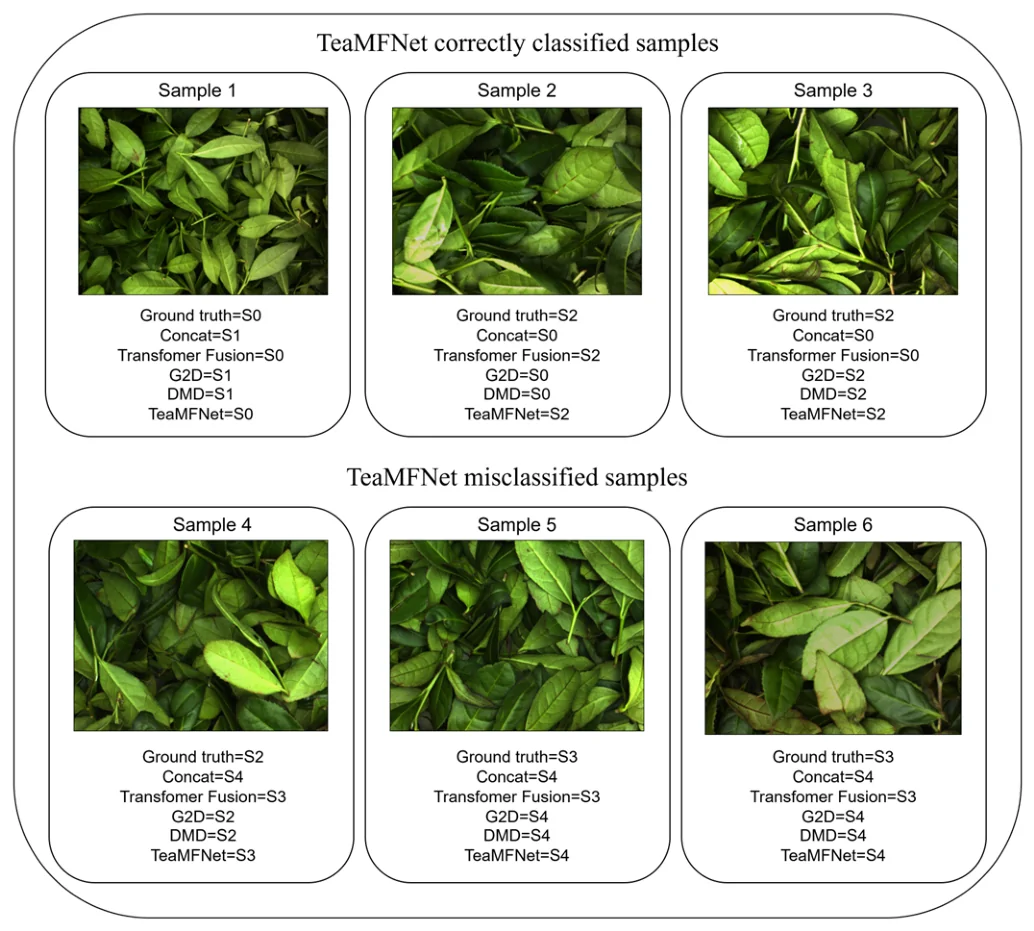
Comparison of recognition results of different fusion models on typical test samples.

Figure 7 compares predictions on representative test samples, with one RGB image displayed per sample for clarity. The upper row presents samples correctly recognized by TeaMFNet but misclassified by some comparison models, whereas the lower row shows those still misclassified by TeaMFNet. For several easily confused samples, one or more comparison models produced incorrect stage predictions, whereas TeaMFNet classified them correctly. This suggests that the effective utilization of complementary RGB–HSI information improves the discrimination of challenging samples. Meanwhile, TeaMFNet still misclassified certain samples, mainly between adjacent or visually similar stages such as S2–S3 and S3–S4. These samples exhibit subtle differences in leaf color, leaf posture, and local reddening, alongside pronounced transitional characteristics and indistinct stage boundaries. Consistent with the confusion matrices, these findings identify adjacent-stage recognition as a major challenge for intelligent Tieguanyin fermentation monitoring.

**Figure 8:**
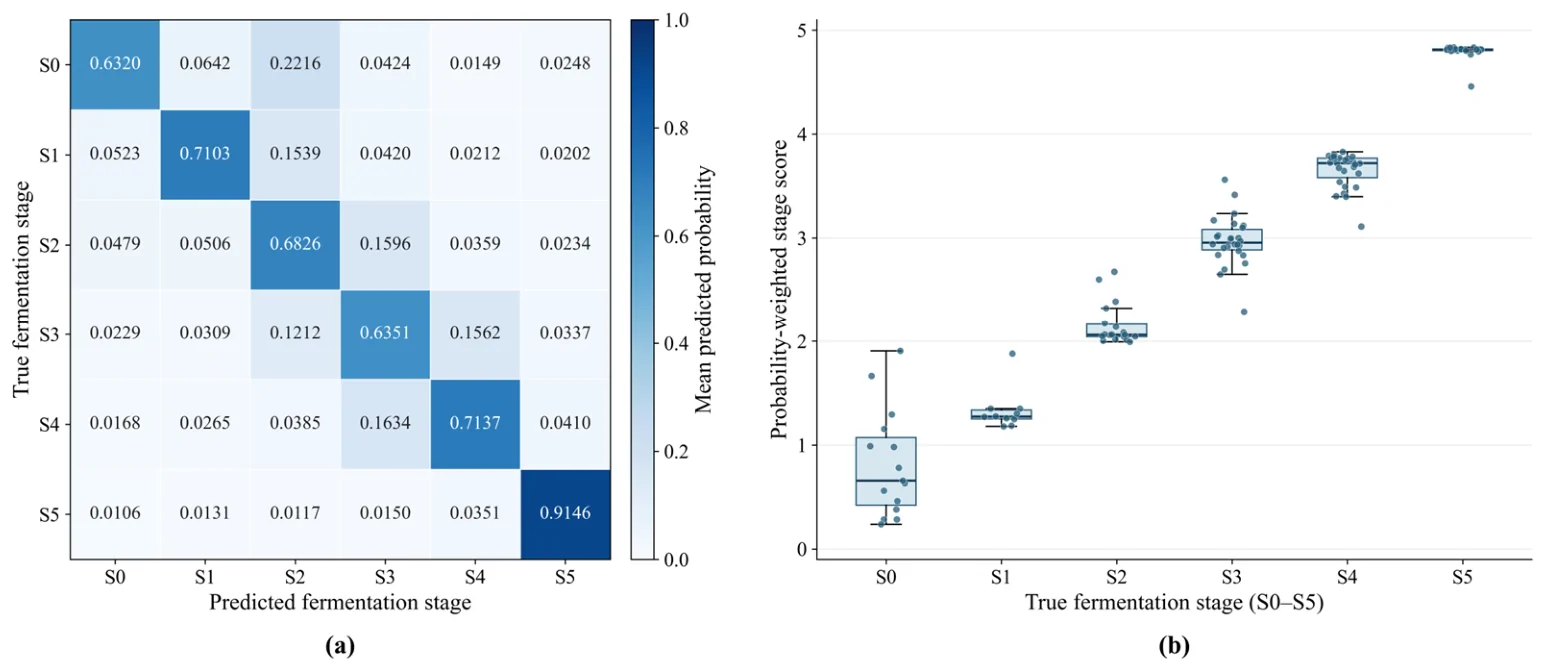
Probability-based analysis of Tieguanyin fermentation stage predictions. (**a**) Heatmap of mean Softmax probability distributions; (**b**) boxplots of probability-weighted stage scores, with individual test samples overlaid as scatter points.

A post-hoc analysis of the 116 test samples examined stage-order information beyond discrete predictions (Figure 8). On average, 88.96% of the predicted probability mass was assigned to the ground-truth stage and its adjacent stages (Figure 8a). Moreover, the two highest-probability classes were adjacent for 103 of the 116 samples (88.79%), suggesting that the predicted probability distributions generally contained stage-order information. Nevertheless, non-adjacent ambiguities remained, particularly for S0, where the mean probability assigned to non-adjacent stages reached 30.37%.

Mean probability-weighted stage scores increased across S0–S5, from 0.818 to 4.795 (Figure 8b). The Spearman correlation coefficient between the scores and the ground-truth stage labels was 0.9759, indicating a strong descriptive association with stage ordering. These results provide supplementary stage-order information; however, given the uncalibrated probabilities, the scores should be interpreted as auxiliary ordinal descriptors rather than measurements of continuous biochemical fermentation degree.

#### 3.1.3. Cross-Source Generalization Evaluation

To evaluate TeaMFNet’s cross-source generalization, a leave-one-source-out evaluation was conducted across three tea gardens. In each of the three rounds, data from two tea gardens were used for model development, while the remaining tea garden was held out exclusively for testing, with each garden tested once. The three sources corresponded to low (504.59 m), medium (582.58 m), and high (859.88 m) local elevation conditions, respectively.

As shown in Table 6, TeaMFNet achieved accuracies of 80.22%, 91.45%, and 74.10% for held-out Gardens A, B, and C, respectively. Compared with the 93.28% accuracy obtained under the random sample-level split, performance decreased particularly for Gardens A and C. These reductions suggest sensitivity to source-dependent variations and indicate that performance on the random split does not fully represent generalization to unseen sources. Broader cross-source validation is therefore required.

### 3.2. Ablation Study Analysis

To evaluate the contributions of BCF, RFA, and SPM to model performance, a feature-concatenation model trained with the MCM strategy was constructed as the baseline. The baseline employed the same RGB and HSI encoders as TeaMFNet and directly concatenated their features for fermentation stage recognition using an MLP classifier. On this basis, BCF, RFA, and SPM were progressively added for ablation analysis (Table 7).

The full model achieved the best performance, with an accuracy of 93.28% and a Macro-F1 score of 93.38%, outperforming the baseline by 5.42 percentage points and 4.80 percentage points, respectively. These results indicate that the joint introduction of BCF, RFA, and SPM effectively improves RGB–HSI bimodal fermentation stage recognition. Specifically, after BCF was introduced, the model’s accuracy increased from 87.86% to 89.48%, and Macro-F1 increased from 88.58% to 90.64%, indicating that bidirectional cross-modal feature updating facilitates complementary information exchange between the RGB and HSI representations. Further incorporating RFA increased accuracy and Macro-F1 to 91.55% and 91.72%, respectively, suggesting that residual correction of the HSI representation using the RGB representation as a discriminative reference improves cross-modal alignment and enables HSI information to contribute more effectively to fusion-based discrimination.

Compared with Baseline + BCF, adding SPM increased accuracy and Macro-F1 to 90.52% and 90.91%, corresponding to improvements of 1.04 percentage points and 0.27 percentage points, respectively. These results suggest a modest benefit from stage prototype constraints for fused-feature discriminability. The full model (TeaMFNet) achieved the best performance, exceeding Baseline + BCF + RFA by 1.73 percentage points and 1.66 percentage points in accuracy and Macro-F1, respectively. This suggests that once RFA aligns the RGB and HSI representations, SPM can further enhance the intra-stage compactness and inter-stage separability of the fused features.

### 3.3. Analysis of Modality Contributions and Complementarity

To assess modality contributions and complementarity, the RGB unimodal model (RGB ResNet50), HSI unimodal model (HSI 3D-CNN), basic feature-concatenation model (Concat Fusion), and TeaMFNet were compared (Table 8). All models used identical paired-sample splits. The RGB and HSI unimodal models used each sample’s three RGB views and corresponding HSI cube, respectively.

The RGB unimodal model achieved an accuracy of 75.29% and a Macro-F1 of 75.34%, outperforming the HSI unimodal model (68.86% and 66.56%, respectively). These results indicate that RGB images directly capture appearance changes, thereby providing relatively strong discriminative information. Although HSI data contain richer spatial–spectral information, their poorer standalone performance may reflect high feature dimensionality and the subtle spectral differences between adjacent stages.

To verify whether RGB images and HSI data provide complementary information, Concat Fusion directly concatenated features from both modalities. It achieved an accuracy of 86.22% and a Macro-F1 of 86.13%, outperforming the RGB unimodal model by 10.93 percentage points and 10.79 percentage points and the HSI unimodal model by 17.36 percentage points and 19.57 percentage points, respectively. These results confirm that adding HSI information to RGB appearance features substantially improves recognition performance, demonstrating the complementarity between the two modalities.

TeaMFNet achieved an accuracy of 93.28% and a Macro-F1 of 93.38%, representing improvements of 7.06 percentage points and 7.25 percentage points over Concat Fusion. These results confirm that although simple feature concatenation can utilize complementary bimodal information, it lacks explicit interaction between global modality representations before final fusion. Through bidirectional cross-modal feature updating, feature alignment, and stage-discriminative constraints, TeaMFNet exploits RGB–HSI complementarity more effectively.

### 3.4. Analysis of Fused Feature Distribution and Separability

#### 3.4.1. Visualization of Fused Feature Distributions

**Figure 9:**
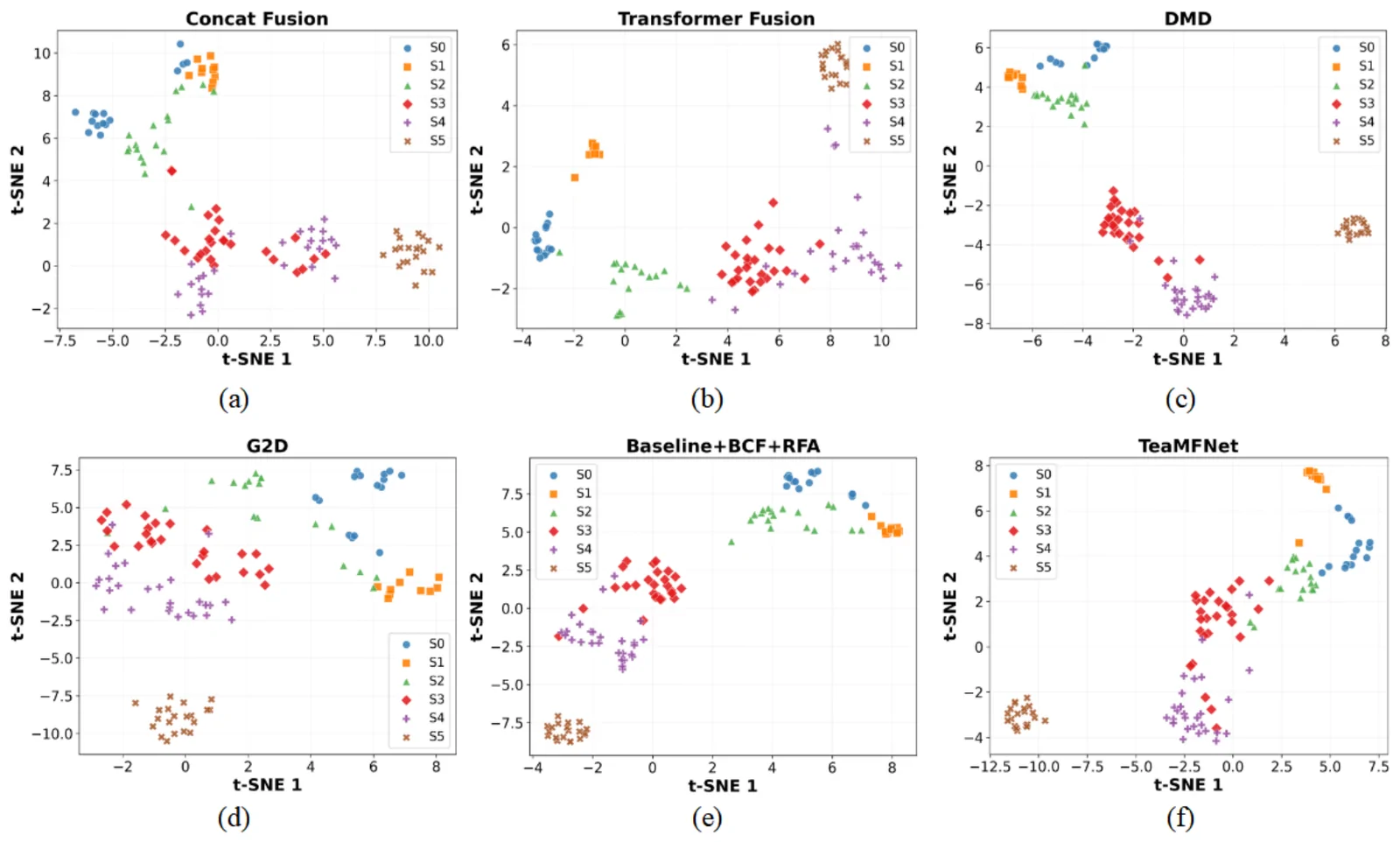
The t-SNE visualization results of fused feature distributions of different models. All results were obtained from models trained with random seed 42. (**a**) Concat Fusion; (**b**) Transformer Fusion; (**c**) DMD; (**d**) G2D; (**e**) Baseline + BCF + RFA; and (**f**) TeaMFNet.

To visualize learned feature distributions, t-SNE projected the high-dimensional fused features of test samples into a two-dimensional space (Figure 9), with different colors denoting different fermentation stages. More discriminative stage features are expected to produce more compact intra-stage clusters and clearer inter-stage separation in the feature space. The analysis included four comparison models—Concat Fusion, Transformer Fusion, DMD, and G2D—along with Baseline + BCF + RFA (the full model without SPM) to examine the effect of stage prototype constraints on feature distributions and inter-stage separability.

Concat Fusion distinguished different fermentation stages to some extent, but samples from several adjacent stages still exhibited overlapping distributions (Figure 9a). In particular, samples around S2, S3, and S4 were closely clustered, indicating that simple feature concatenation remained limited in characterizing stage boundaries. Transformer Fusion and DMD showed improved intra-stage clustering and inter-stage separation over Concat Fusion (Figure 9b,c), suggesting that attention-based interaction and modality disentanglement can enhance the representational capability of fused features to some extent. Samples from several stages in G2D still exhibited relatively scattered distributions, with overlap between certain stages (Figure 9d), suggesting that although modality balancing alleviates strong-modality dominance, it remains limited in capturing the subtle differences between adjacent stages. In Figure 9e, Baseline + BCF + RFA formed more distinct stage clusters than the feature-concatenation baseline, indicating that bidirectional cross-modal feature updating and residual feature alignment enhance the stage discriminability of RGB–HSI fused features. However, some intra-stage dispersion remained at certain stages.

The fused features learned by TeaMFNet (Figure 9f) exhibited a clear stage-wise clustering pattern. The six stages (S0–S5) formed relatively distinct clusters in the two-dimensional space, suggesting that the full model learned fused features with stronger stage discriminability. Compared with Baseline + BCF + RFA, incorporating SPM improved intra-stage compactness and inter-stage separability, supporting its contribution to the stage-wise organization of the fused features.

#### 3.4.2. Analysis of Fused Feature Separability

To quantify stage-wise fused-feature separability, the average intra-class distance, average inter-class distance, and the ratio of the two distances were computed for the test-set features. Smaller intra-class distances indicate that samples from the same fermentation stage are more tightly clustered in the feature space, whereas a larger average inter-class distance indicates greater separation between different fermentation stages. A larger inter-class-to-intra-class ratio therefore reflects stronger overall feature separability.

As shown in Table 9, the models differed across the distance-based metrics. Transformer Fusion achieved the smallest average intra-class distance of 3.31 and the highest inter-class-to-intra-class ratio of 4.47, indicating relatively compact within-stage clustering. However, its average inter-class distance was only 14.81, which was lower than those of DMD, Concat Fusion, Baseline + BCF + RFA, and TeaMFNet, indicating limited separation between stages. Therefore, no single distance metric can fully reflect the final recognition performance of a model, and these metrics should be interpreted together with the classification results for a comprehensive evaluation.

Compared with Concat Fusion, Baseline + BCF + RFA increased the average inter-class distance from 20.81 to 23.34, indicating that bidirectional cross-modal feature updating and residual feature alignment enhance between-stage separation. However, it also exhibited a relatively large average intra-class distance of 7.68, resulting in an inter-class-to-intra-class ratio of only 3.04. This suggests that cross-modal feature updating and feature alignment alone are insufficient to fully enhance the compactness of samples within the same fermentation stage. After adding SPM, TeaMFNet reduced the average intra-class distance from 7.68 to 6.58, increased the average inter-class distance from 23.34 to 25.83, and raised the inter-class-to-intra-class ratio from 3.04 to 3.93. These results indicate that stage prototype constraints enhance both within-stage compactness and between-stage separation.

Overall, although TeaMFNet did not achieve the best value for every distance-based metric, it obtained the largest average inter-class distance and a relatively high inter-class-to-intra-class ratio, together with the highest accuracy and Macro-F1. These results indicate that TeaMFNet achieves more stable fermentation stage recognition while maintaining strong stage-wise feature separability.

### 3.5. Spectral Interpretability Analysis

**Figure 10:**
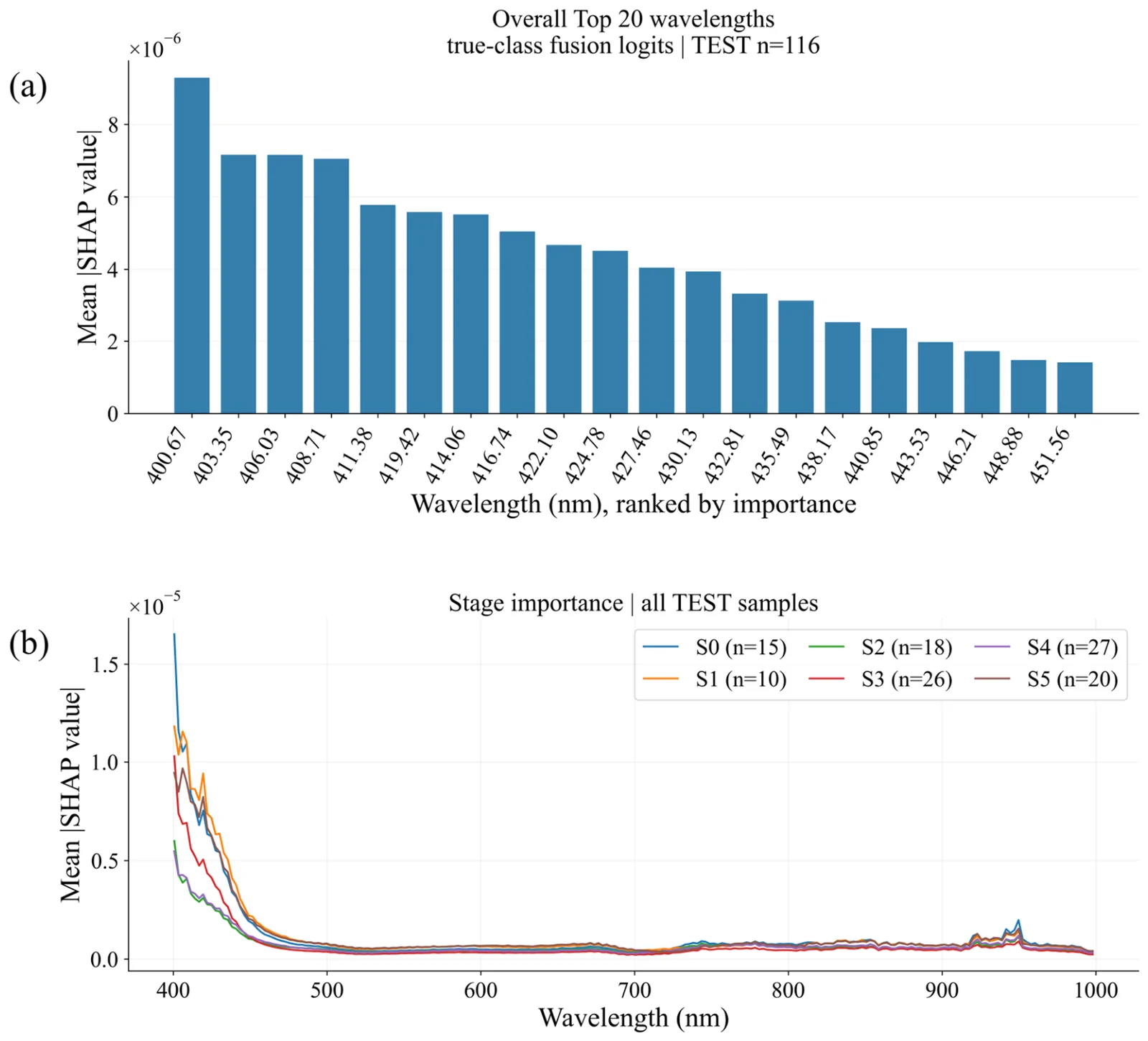
GradientSHAP-based spectral attribution analysis of TeaMFNet. (**a**) Overall importance ranking of the top 20 wavelengths across the test set; (**b**) stage-wise spectral importance profiles.

The GradientSHAP results identified the spectral bands contributing to TeaMFNet predictions. Figure 10a shows that the top 20 wavelengths ranked by mean absolute SHAP values were concentrated within 400.67–451.56 nm, with 400.67 nm exhibiting the highest attribution. Figure 10b shows broadly similar wavelength importance profiles across S0–S5 but differing attribution magnitudes, indicating stage-specific differences in wavelength contributions to classification outputs.

Table 10 summarizes the five wavelengths with the highest mean absolute GradientSHAP values per stage. The selected wavelengths were consistently concentrated within the range of 400.67–419.42 nm across all six stages. Specifically, 400.67 nm ranked first for S0–S4, whereas 406.03 nm exhibited the highest attribution for S5. Four wavelengths, namely 400.67, 403.35, 406.03, and 408.71 nm, appeared among the top five for every stage. The ranking order was identical for S0, S2, and S3. S4 contained the same five wavelengths but in a different order, whereas S1 and S5 included 419.42 nm instead of 411.38 nm. These findings indicate that TeaMFNet relied on broadly shared short-wavelength spectral information across fermentation stages, with differences in attribution magnitude and, for some stages, wavelength ranking rather than entirely distinct sets of important wavelengths.

From a spectroscopic and food chemistry perspective, the relatively high attribution observed in the 400–450 nm region may be associated with fermentation-related changes in leaf pigments and phenolic compounds. Previous research on Tieguanyin fermentation stage recognition using RGB and hyperspectral features identified wavelengths near 413 and 416 nm as important predictors [21], which fall within the high-attribution region observed in this study. Similarly, a study integrating visible–near-infrared spectroscopy and image-processing technologies for monitoring oolong tea fermentation reported a spectral feature near 450 nm [45]. These findings suggest that the short-wavelength visible region may contain information relevant to fermentation stage discrimination.

The potential chemical relevance of this region can be considered from two perspectives. First, chlorophyll a and chlorophyll b exhibit strong absorption in the blue-violet region, with reported absorption maxima near 430 and 453 nm, respectively, while carotenoids exhibit broader absorption across approximately 400–500 nm [46]. Changes in leaf pigment composition during tea processing may therefore affect the optical properties of tea leaves in this spectral region. Second, the enzymatic oxidation and polymerization of catechins during oolong tea processing can generate theaflavins and other phenolic oxidation products [47], potentially contributing to changes in leaf color and visible optical properties. Together, these processes provide plausible, testable explanations for the potential relevance of short-wavelength spectral information to fermentation stage recognition. Their relationships with the observed attribution patterns could be further investigated through paired physicochemical measurements of leaf pigments and phenolic compounds alongside hyperspectral observations. The highest-attribution wavelengths (400.67–419.42 nm) lie near the lower boundary of the FX10 spectral range, where the signal-to-noise ratio may be relatively low, and below the reported chlorophyll absorption maxima near 430 and 453 nm. These attributions may partly reflect instrumental boundary effects or preprocessing sensitivity and do not directly establish a chlorophyll-related mechanism. Their biochemical relevance therefore requires further validation.

### 3.6. Computational Complexity and Inference Efficiency

The computational complexity and inference efficiency of TeaMFNet were compared with those of Concat Fusion, DMD, and G2D using an NVIDIA GeForce RTX 4090 GPU (Table 11). TeaMFNet contained 25.79 M parameters, representing an increase of approximately 5.99% compared with Concat Fusion (24.33 M). The FLOPs of the two models were 51.2814 G and 51.2785 G, respectively, indicating a relatively small difference in theoretical computational cost. This suggests that the shared RGB and HSI feature extraction components account for a substantial proportion of the overall computation. In terms of inference efficiency, TeaMFNet required an average GPU inference time of 8.47 ms per paired sample, compared with 7.53 ms for Concat Fusion, corresponding to an increase of 12.48%. Meanwhile, peak GPU memory consumption increased from 380.93 MB to 386.77 MB. Compared with DMD, TeaMFNet had fewer parameters and slightly lower FLOPs, although its GPU inference latency was higher.

The computational overhead should also be considered in relation to the recognition performance of fusion models. As reported in Table 4, TeaMFNet achieved an accuracy of 93.28%, compared with 86.22% for Concat Fusion, representing an improvement of 7.06 percentage points. This improvement was accompanied by an increase of approximately 5.99% in model parameters, while GPU inference latency increased from 7.53 ms to 8.47 ms per paired sample. In contrast, the differences in FLOPs and peak GPU memory consumption were relatively small. These results indicate that TeaMFNet achieved improved fermentation stage recognition performance with additional inference time but a relatively small increase in theoretical computational cost and memory requirements.

It should be noted that inference latency was measured under laboratory conditions. The computational performance of TeaMFNet has not yet been evaluated on industrial edge hardware. Therefore, although the results provide a quantitative assessment of its computational requirements, the real-time performance and practical deployment feasibility of TeaMFNet in industrial tea-processing environments require further validation.

## 4. Conclusions

Given the progressive nature of Tieguanyin fermentation and the subtle differences between adjacent stages, accurate fermentation stage recognition remains challenging, particularly when relying on a single modality. To address this challenge, this study developed TeaMFNet to enhance cross-modal information exchange, feature alignment, and stage discriminability.

Experiments on a self-constructed RGB–HSI bimodal dataset demonstrated RGB–HSI complementarity. The RGB and HSI unimodal models achieved accuracies of 75.29% and 68.86%, respectively, while Concat Fusion improved the accuracy to 86.22%. TeaMFNet achieved an accuracy of 93.28% and a Macro-F1 score of 93.38%, showing competitive recognition performance among the evaluated fusion models. These results indicate that TeaMFNet effectively integrates RGB appearance features and HSI spatial–spectral information to improve stage recognition under the investigated conditions. Ablation experiments supported the complementary contributions of BCF, RFA, and SPM to feature interaction, alignment, and stage discrimination. Feature separability analysis showed that SPM reduced the average intra-class distance from 7.68 to 6.58 and increased the average inter-class distance from 23.34 to 25.83. Additionally, GradientSHAP analysis identified relatively high spectral attribution in the 400–450 nm region across all six fermentation stages, highlighting spectral information utilized by TeaMFNet.

This study still has several limitations. Firstly, although the leave-one-source-out evaluation provided additional evidence of cross-source recognition performance, performance varied among the three tea gardens. Also, since each elevation category was represented by only one tea garden and all samples were collected within the same local production area and a limited harvesting period, broader generalizability across different harvesting seasons and production environments remains to be validated. Secondly, the study focused on recognizing representative processing stages rather than estimating continuous fermentation degree. Regression was not investigated because quantitative physicochemical reference measurements were unavailable. Ordinal classification, which could exploit the inherent ordering of the six stages, was also not directly compared with the proposed classification framework. Thirdly, the biochemical relevance of GradientSHAP-attributed regions requires direct physicochemical validation, particularly given potential instrumental and preprocessing effects near the lower spectral boundary. In addition, inference efficiency was evaluated on an NVIDIA GeForce RTX 4090 GPU under laboratory conditions, without accounting for the complete data acquisition and preprocessing workflow. The real-time performance of TeaMFNet on industrial edge hardware therefore remains to be assessed. Future research will focus on broader cross-source and cross-season validation, quantitative physicochemical measurements, ordinal classification and regression-based approaches, and evaluation of end-to-end processing efficiency under practical tea-processing conditions.

## Figures and Tables

**Table 1:** Representative RGB images and hyperspectral data at different fermentation stages.

Stage	HSI Cube	RGB View 1	RGB View 2	RGB View 3
S0	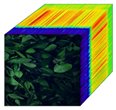	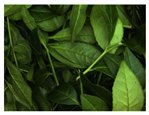	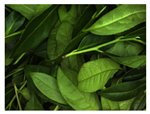	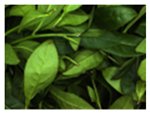
S1	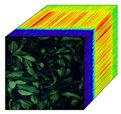	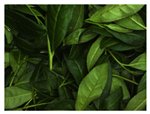	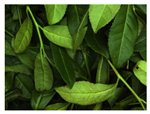	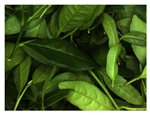
S2	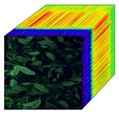	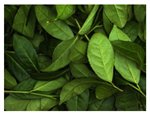	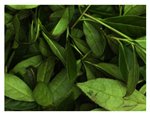	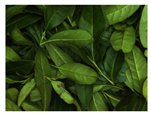
S3	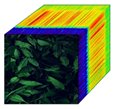	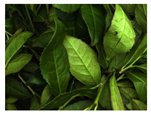	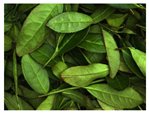	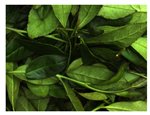
S4	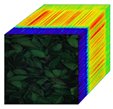	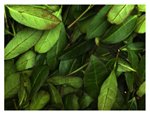	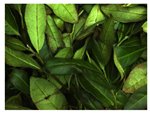	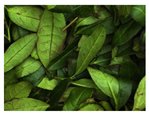
S5	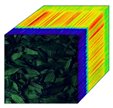	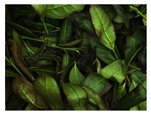	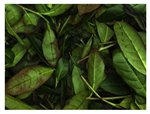	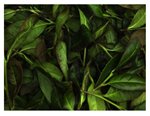

**Table 2:** Number of valid samples at different fermentation stages.

Stage	Number of HSI Samples	Number of RGB Images
S0	86	258
S1	59	177
S2	103	309
S3	158	474
S4	169	507
S5	120	360
Total	695	2085

**Table 3:** Model training parameter settings.

Parameter	Value
Input RGB image size	224 × 224
HSI input size	1 × 224 × 96 × 96
Batch size	4
Number of training epochs	20
Optimizer	AdamW
Initial learning rate	2 × 10^−5^
Weight decay	1 × 10^−4^
MCM stage threshold, $\tau_{g}$	10
Alignment loss weight, $\lambda_{align}$	0.01
Prototype loss weight, $\lambda_{proto}$	0.1
Prototype scaling factor, s	16
Random seeds	1, 2 and 42

**Table 4:** Comparison of recognition performance of different models on the test set.

Model	Input Modality	Accuracy (%)	Macro-F1 (%)
Concat Fusion	RGB + HSI	86.22 ± 0.36	86.13 ± 0.44
LMF	RGB + HSI	87.22 ± 0.85	87.01 ± 0.96
Transformer Fusion	RGB + HSI	88.31 ± 0.64	89.47 ± 0.56
DMD	RGB + HSI	92.14 ± 0.21	91.47 ± 0.56
TCPFMC	RGB + HSI	85.34 ± 0.53	83.35 ± 0.23
EMOE	RGB + HSI	78.41 ± 0.72	68.47 ± 0.35
G2D	RGB + HSI	88.58 ± 0.77	89.38 ± 0.28
MultiEMO	RGB + HSI	87.56 ± 0.63	88.44 ± 0.54
TeaMFNet	RGB + HSI	93.28 ± 0.70	93.38 ± 0.22

**Table 5:** Recognition accuracy (%) of different fusion models across fermentation stages. All results were obtained from models trained with random seed 42.

Model	S0	S1	S2	S3	S4	S5
Concat Fusion	86.67	100.00	66.67	76.92	96.30	100.00
Transformer Fusion	100.00	100.00	83.33	100.00	59.26	100.00
DMD	80.00	100.00	94.44	88.46	92.59	100.00
G2D	93.33	100.00	72.22	80.77	92.59	100.00
TeaMFNet	86.67	90.00	94.44	84.62	100.00	100.00

**Table 6:** Recognition performance of TeaMFNet under leave-one-source-out evaluation. The results correspond to a single training run with random seed 42 for each held-out source.

Tea Garden Source	Mean Elevation (m)	Accuracy (%)	Macro-F1 (%)
A (Low elevation)	504.59	80.22	76.83
B (Medium elevation)	582.58	91.45	90.81
C (High elevation)	859.88	74.10	72.33

**Table 7:** Recognition performance of ablation experiments.

Model	BCF	RFA	SPM	Accuracy (%)	Macro-F1 (%)
Baseline				87.86 ± 0.94	88.58 ± 0.68
Baseline + BCF	√			89.48 ± 0.68	90.64 ± 0.88
Baseline + BCF + RFA	√	√		91.55 ± 0.75	91.72 ± 1.44
Baseline + BCF + SPM	√		√	90.52 ± 0.61	90.91 ± 0.49
TeaMFNet	√	√	√	93.28 ± 0.70	93.38 ± 0.22

**Table 8:** Comparison of recognition performance between unimodal and bimodal models.

Model	Input Modality	Accuracy (%)	Macro-F1 (%)
RGB ResNet50	RGB	75.29 ± 0.36	75.34 ± 0.45
HSI 3D-CNN	HSI	68.86 ± 0.12	66.56 ± 0.23
Concat Fusion	RGB + HSI	86.22 ± 0.36	86.13 ± 0.44
TeaMFNet	RGB + HSI	93.28 ± 0.70	93.38 ± 0.22

**Table 9:** Comparison of intra-class distance, inter-class distance, and their ratio of fused features among different models. All results were obtained from models trained with random seed 42.

Model	Average Intra-Class Distance	Average Inter-Class Distance	Inter-Class-to-Intra-Class Ratio
Concat Fusion	6.63	20.81	3.13
Transformer Fusion	3.31	14.81	4.47
DMD	5.27	18.72	3.55
G2D	3.53	10.16	2.87
Baseline + BCF + RFA	7.68	23.34	3.04
TeaMFNet	6.58	25.83	3.93

**Table 10:** Top five wavelengths ranked by mean absolute GradientSHAP values per stage.

Stage	Rank 1 (nm)	Rank 2 (nm)	Rank 3 (nm)	Rank 4 (nm)	Rank 5 (nm)
S0	400.67	403.35	408.71	406.03	411.38
S1	400.67	406.03	408.71	403.35	419.42
S2	400.67	403.35	408.71	406.03	411.38
S3	400.67	403.35	408.71	406.03	411.38
S4	400.67	406.03	403.35	408.71	411.38
S5	406.03	400.67	408.71	403.35	419.42

**Table 11:** Comparison of computational complexity and inference efficiency among different fusion models.

Model	Parameters (M)	FLOPs (G)	GPU Latency (ms)	Peak GPU Memory (MB)
Concat Fusion	24.33	51.2785	7.53	380.93
DMD	26.31	51.2847	7.74	388.87
G2D	24.43	51.2787	7.62	381.34
TeaMFNet	25.79	51.2814	8.47	386.77

## Data Availability

The data supporting the findings of this study are available from the corresponding author upon reasonable request.
